# Author Correction: A quinary WTaCrVHf nanocrystalline refractory high-entropy alloy withholding extreme irradiation environments

**DOI:** 10.1038/s41467-023-39294-8

**Published:** 2023-06-13

**Authors:** O. El Atwani, H. T. Vo, M. A. Tunes, C. Lee, A. Alvarado, N. Krienke, J. D. Poplawsky, A. A. Kohnert, J. Gigax, W.-Y. Chen, M. Li, Y. Q. Wang, J. S. Wróbel, D. Nguyen-Manh, J. K. S. Baldwin, O. U. Tukac, E. Aydogan, S. Fensin, E. Martinez

**Affiliations:** 1 Materials Science and Technology Division, Los Alamos National Laboratory, Los Alamos, NM USA; 2 Center for Integrated Nanotechnology, Los Alamos National Laboratory, Los Alamos, NM USA; 3 Department of Materials and Mechanical Engineering, Auburn University, Auburn, AL USA; 4 Theoretical Division, Los Alamos National Laboratory, Los Alamos, NM USA; 5 Departments of Mechanical Engineering and Materials Science and Engineering, Clemson University, Clemson, SC USA; 6 Materials Science and Engineering, University of Wisconsin-Madison, Madison, WI USA; 7 Materials Science and Technology Division, Oak Ridge National Laboratory, Oak Ridge, TN USA; 8 Division of Nuclear Engineering, Argonne National Laboratory, Lemon, IL USA; 9 Faculty of Materials Science and Engineering, Warsaw University of Technology, ul. Wołoska, 02−507 Warsaw, Poland; 10 Culham Center for Fusion Energy, United Kingdom Atomic Energy Authority, Abingdon, OX14 3DB UK; 11 Department of Materials, University of Oxford, Oxford, OX1 3PH UK; 12 Metallurgical and Materials Engineering, Middle East Technical University, Ankara, Turkey

**Keywords:** Metals and alloys, Power stations

Correction to: *Nature Communications* 10.1038/s41467-023-38000-y, published online 02 May 2023

The original version of this Article contained an error in the affiliation of the 4^th^ author, C. Lee from ‘Department of Materials and Mechanical Engineering, Auburn University, Auburn, AL, USA’, which was incorrectly given as ‘Department of Materials and Mechanical Engineering, Auburn University, Montgomery, AL, USA’. The error has been corrected in both the PDF and HTML versions of the Article.

