# Supplementary material for: A quinary WTaCrVHf nanocrystalline refractory high-entropy alloy withholding extreme irradiation environments
Source: Nat Commun. 2023 May 2;14:2516. doi: 10.1038/s41467-023-38000-y (PMC10154406; doi:10.1038/s41467-023-38000-y)
Supplement: Supplementary file 1 — Supplementary Information [file 41467_2023_38000_MOESM1_ESM.pdf]

Supplementary Information for "A quinary WTaCrVHf nanocrystalline refractory high-entropy alloy withholding extreme irradiation environments."

Supplementary Figures & Tables

The dpa and He implantation profiles were calculated using the Kinchin-Pease model in the Stopping & Range of Ions in Matter (SRIM) Monte Carlo computer simulation code (version 2013)[1] and 40 eV [2] was taken as the displacement threshold energy for all elements.

1 MeV Kr^{+2}

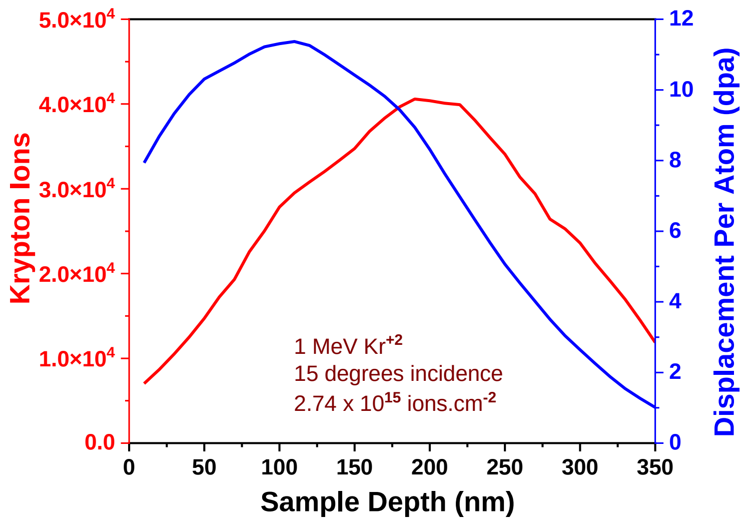

Supplementary Figure 1: Displacement per damage (dpa) and Ions distributions for 1 MeV Kr^{+} irradiation**.**

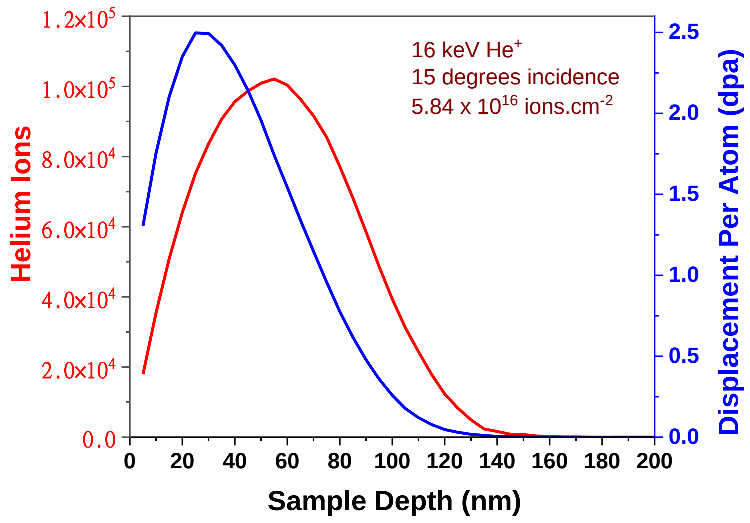

Supplementary Figure 2: Displacement per damage (dpa) and Ions distributions for 16 keV He^{+} implantation.

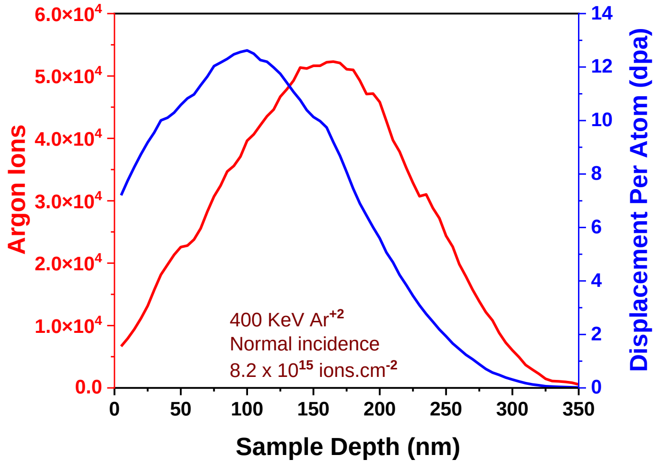

Supplementary Figure 3: Displacement per damage (dpa) and Ions distributions for 400 keV Ar^{+2} implantation.

Thermophysical properties were calculated for W-Ta-Cr-V and W-Ta-Cr-Fe systems. The results are in Table 1. The atomic radii and enthalpy used in the calculations are taken from refs.[3,4]

Supplementary Table 1: Thermophysical parameter calculations for different compositions of the W-Ta-Cr-V and W-Ta-Cr-Fe systems

	1		2		3		4		Density	VEC	ΔS mix	Tm (K)	δ[1]	ΔH mix[2]	Ω
A	Cr	25	V	25	Ta	25	W	25	12.30	5.5	-11.53	2837	4.96	-4.19	7.80
A	Cr	15	V	35	Ta	15	W	35	12.45	5.5	-10.84	2878	4.03	-2.95	10.59
A	Cr	15	V	35	Ta	35	W	15	11.93	5.3	-10.84	2797	4.79	-3.91	7.75
A	Cr	15	V	15	Ta	35	W	35	14.56	5.5	-10.84	3099	4.55	-5.33	6.30
A	Cr	35	V	15	Ta	15	W	35	12.67	5.7	-10.84	2877	4.94	-3.15	9.92
A	Cr	35	V	15	Ta	35	W	15	12.15	5.5	-10.84	2796	5.75	-5.29	5.73
A	Cr	35	V	35	Ta	15	W	15	10.04	5.5	-10.84	2575	4.75	-3.19	8.75
B	Cr	25	Fe	25	Ta	25	W	25	12.74	6.25	-11.53	2744	6.05	-7.34	4.31
B	Cr	15	Fe	15	Ta	35	W	35	14.82	5.95	-10.84	3043	5.56	-8.03	4.11
B	Cr	15	Fe	35	Ta	15	W	35	13.07	6.55	-10.84	2748	5.64	-5.38	5.54
B	Cr	15	Fe	35	Ta	35	W	15	12.55	6.35	-10.84	2667	6.51	-10.42	2.77
B	Cr	35	Fe	15	Ta	15	W	35	12.93	6.15	-10.84	2821	5.52	-4.13	7.41
B	Cr	35	Fe	15	Ta	35	W	15	12.41	5.95	-10.84	2740	6.38	-8.02	3.70
B	Cr	35	Fe	35	Ta	15	W	15	10.66	6.55	-10.84	2445	5.62	-5.69	4.65
			Single phase criteria:										6.6	-15< ΔH mix < (5)	1.1

The W-Ta-Cr-V system demonstrated stable single BCC phases. The VEC values for the W-Ta-Cr-Fe are high (which still indicating a single BCC phase). However, the phase stability diagrams show no stable single phase but mostly intermetallics. WTaCrFe showed Laves phase. The laves phase contains mostly Fe and Ta. Minimizing Ta and Fe (e.g. Cr35Fe15Ta15W35) still shows intermetallics. Examples are given below:

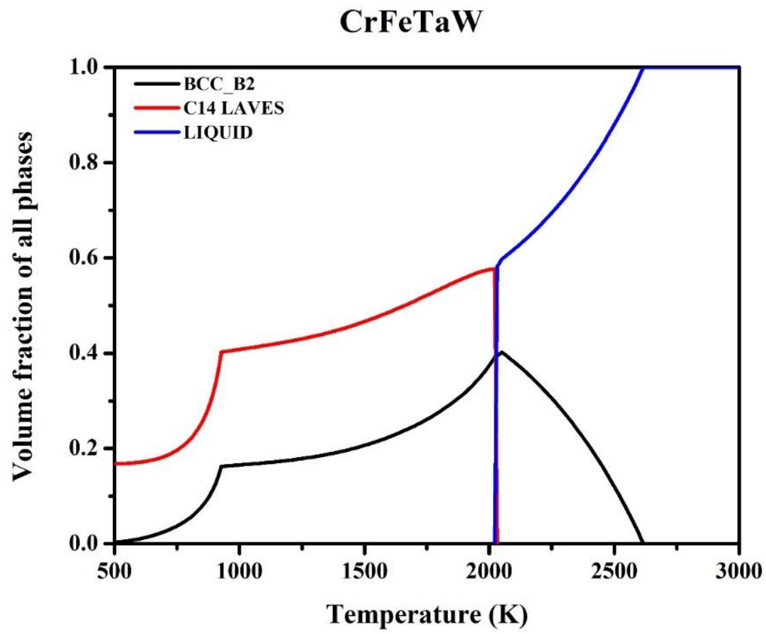

Supplementary Figure 4: Calphad phase stability diagram for CrFeTaW

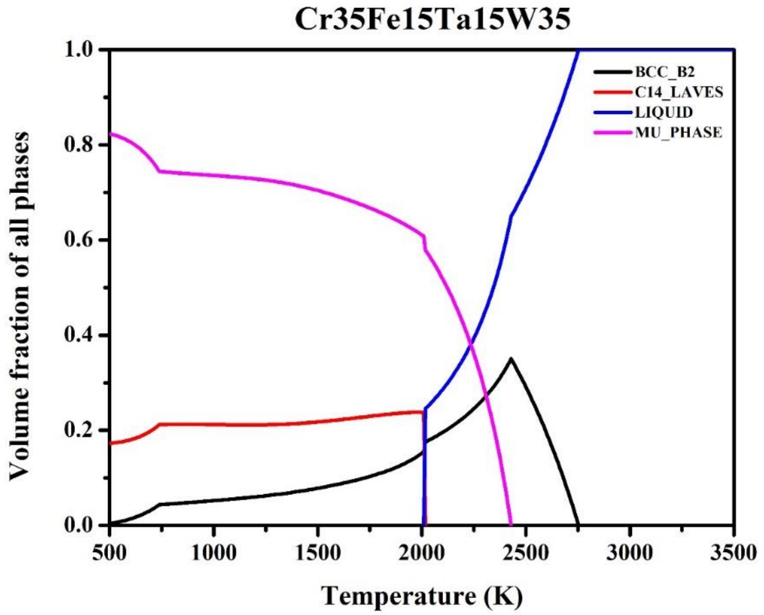

Supplementary Figure 5: Calphad phase stability diagram for Cr35Fe15Ta15W35

Two options are available for possible enhanced ductility

A- One option is using electron theory and decreasing the VEC under 4,4, so that elongation between 5-15% can be achieved (as discussed in the manuscript). However, for this purpose group 4 elements are required (Ti, Zr, Hf).

B- Second option is increasing VEC up to 6,87 so that it has FCC+BCC phase instead of single brittle BCC (discussed in the manuscript).

For the second option (B), compositions are changed in the Cr, Fe, Ta, W to maximize VEC. For this purpose, Fe has to be maximized to ~50%. Alloys 1, 2 and 3 possess the intended VEC numbers. The single-phase criteria are also satisfied (Supplementary Table 2). However, CALPHAD shows intermetallics for these compositions. In addition, the melting points of these compositions are low.

Supplementary Table 2: Thermophysical parameter calculations for the W-Ta-Cr-Fe system with the goal of maximizing the VEC.

	1		2		3		4		Density	VEC	ΔS mix	Tm (K)	δ[1]	ΔH mix[2]	Ω
	Cr	20	Fe	40	Ta	5	W	35	12.16	6.75	-10.02	2618	5.07	-2.20	11.92
1	Cr	10	Fe	50	Ta	5	W	35	12.23	6.95	-9.10	2581	5.12	-2.33	10.09
2	Cr	20	Fe	50	Ta	5	W	25	11.02	6.95	-9.68	2430	4.90	-2.54	9.28
3	Cr	30	Fe	50	Ta	5	W	15	9.81	6.95	-9.50	2278	4.48	-2.82	7.67

The design continued with option A. For this purpose, Hf was added to the W-Ta-Cr-V system. Hf has to be minimized to fit the solid phase criteria and to decrease the radioactivity of the materials. In addition, the trial in compositions is also performed to keep high melting points values. Supplementary Table 3 summarizes the results. 11 compositions satisfied the single-phase criteria and 10 of them were with 5%Hf.

Supplementary Table 3: Thermophysical parameter calculations for the W-Ta-Cr-V-Hf system with the goal of maximizing the VEC. Solid green squares indicate single phase criteria are satisfied.

	Cr	V	Hf	Ta	W	Density	VEC	ΔS mix	Tm (K)	δ[1]	ΔH mix[2]	Ω	solid solution
	35	35	20	5	5	9.11	5.2	-11.28	2378	8.96	-4.86	5.51	no
4	35	35	5	20	5	9.61	5.35	-11.28	2495	6.51	-4.12	6.83	yes
5	35	35	5	5	20	10	5.5	-11.28	2556	5.93	-2.76	10.44	yes
	35	20	35	5	5	10.19	5.05	-11.28	2426	10.29	-6.31	4.34	no
	35	20	5	35	5	11.19	5.35	-11.28	2661	6.9	-4.02	7.47	no
6	35	20	5	5	35	11.97	5.65	-11.28	2783	6.01	-2.67	11.77	yes
	35	5	35	20	5	11.77	5.05	-11.28	2592	9.92	-5.3	5.52	no
	35	5	35	5	20	12.16	5.2	-11.28	2653	10.02	-6.96	4.3	no
	35	5	20	35	5	12.27	5.2	-11.28	2710	8.79	-3.75	8.14	no
	35	5	20	5	35	13.05	5.5	-11.28	2831	8.68	-5.42	5.89	no
	35	5	5	35	20	13.17	5.5	-11.28	2888	6.8	-4.04	8.06	no
7	35	5	5	20	35	13.56	5.65	-11.28	2949	6.47	-4.04	8.22	yes
	20	35	35	5	5	10.03	4.9	-11.28	2426	9.62	-4.96	5.51	no
8	20	35	5	35	5	11.03	5.2	-11.28	2662	6.26	-4.94	6.07	yes
9	20	35	5	5	35	11.81	5.5	-11.28	2783	5.46	-2.27	13.84	yes

	**20**	**5**	**35**	**35**	**5**	**13.19**	**4.9**	**-11.28**	**2759**	**8.53**	**-3.11**	**9.99**	**no**
	**20**	**5**	**35**	**5**	**35**	**13.97**	**5.2**	**-11.28**	**2880**	**8.95**	**-6.48**	**5.01**	**no**
**10**	**20**	**5**	**5**	**35**	**35**	**14.97**	**5.5**	**-11.28**	**3115**	**5.86**	**-5.19**	**6.77**	**yes**
	**5**	**35**	**35**	**20**	**5**	**11.45**	**4.75**	**-11.28**	**2593**	**8.29**	**-3.63**	**8.06**	**no**
	**5**	**35**	**35**	**5**	**20**	**11.84**	**4.9**	**-11.28**	**2654**	**8.49**	**-3.96**	**7.55**	**no**
	**5**	**35**	**20**	**35**	**5**	**11.95**	**4.9**	**-11.28**	**2711**	**7.2**	**-4.35**	**7.03**	**no**
	**5**	**35**	**20**	**5**	**35**	**12.73**	**5.2**	**-11.28**	**2832**	**7.29**	**-3.37**	**9.48**	**no**
**11**	**5**	**35**	**5**	**35**	**20**	**12.84**	**5.2**	**-11.28**	**2889**	**5.21**	**-5.58**	**5.84**	**yes**
**12**	**5**	**35**	**5**	**20**	**35**	**13.23**	**5.35**	**-11.28**	**2950**	**4.98**	**-4.26**	**7.8**	**yes**
	**5**	**20**	**35**	**35**	**5**	**13.03**	**4.75**	**-11.28**	**2759**	**7.51**	**-2.78**	**11.17**	**no**
	**5**	**20**	**35**	**5**	**35**	**13.81**	**5.05**	**-11.28**	**2880**	**8.07**	**-4.82**	**6.73**	**no**
**13**	**5**	**20**	**5**	**35**	**35**	**14.81**	**5.35**	**-11.28**	**3116**	**4.88**	**-5.81**	**6.05**	**yes**
	**5**	**5**	**35**	**35**	**20**	**15**	**4.9**	**-11.28**	**2986**	**6.93**	**-3.76**	**8.95**	**no**
	**5**	**5**	**35**	**20**	**35**	**15.39**	**5.05**	**-11.28**	**3047**	**7.3**	**-5.46**	**6.29**	**no**
**14**	**5**	**5**	**20**	**35**	**35**	**15.89**	**5.2**	**-11.28**	**3164**	**6.29**	**-5.55**	**6.43**	**yes**

From the 11 compositions in table 3 that satisfied the single phase criteria, only 3 compositions demonstrated a large single phase region in CALPHAD.

The phase stability diagrams are shown below:

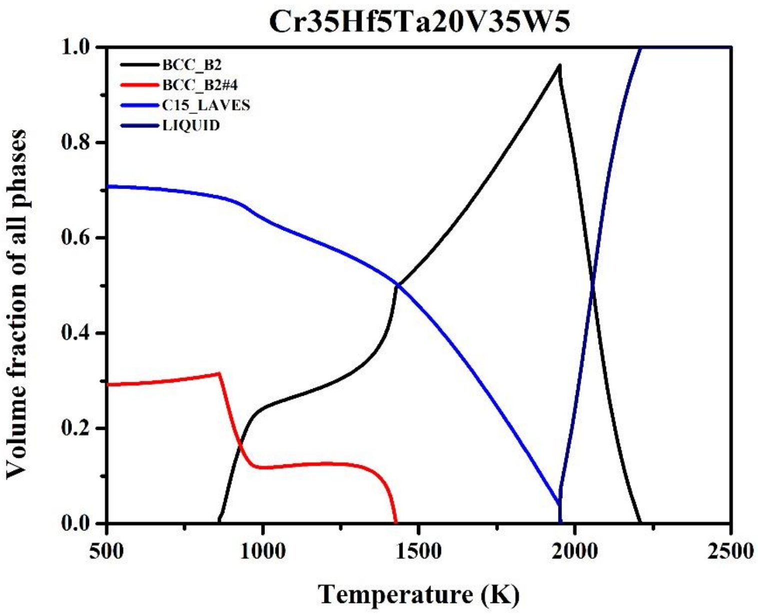

Supplementary Figure 6: Calphad phase stability diagram for Cr35Hf5Ta20V35W5

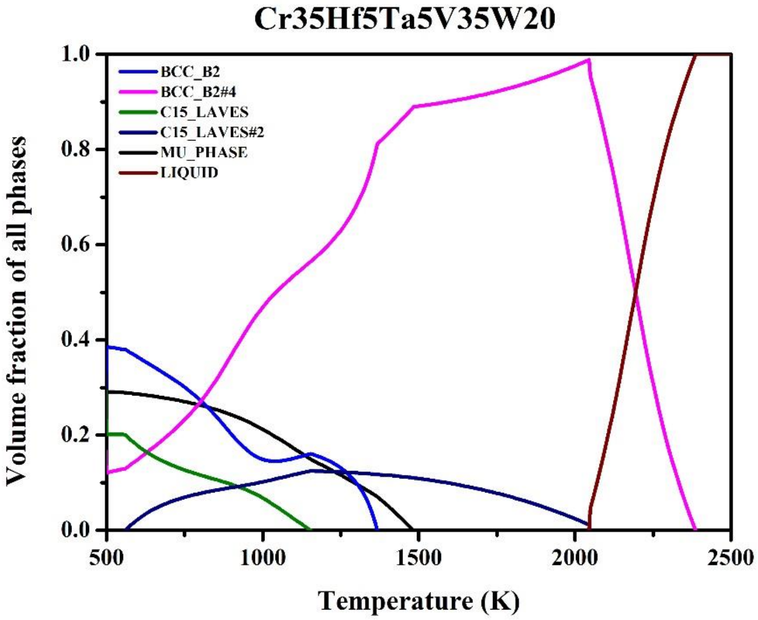

Supplementary Figure 7: Calphad phase stability diagram for Cr35Hf5Ta5V35W20

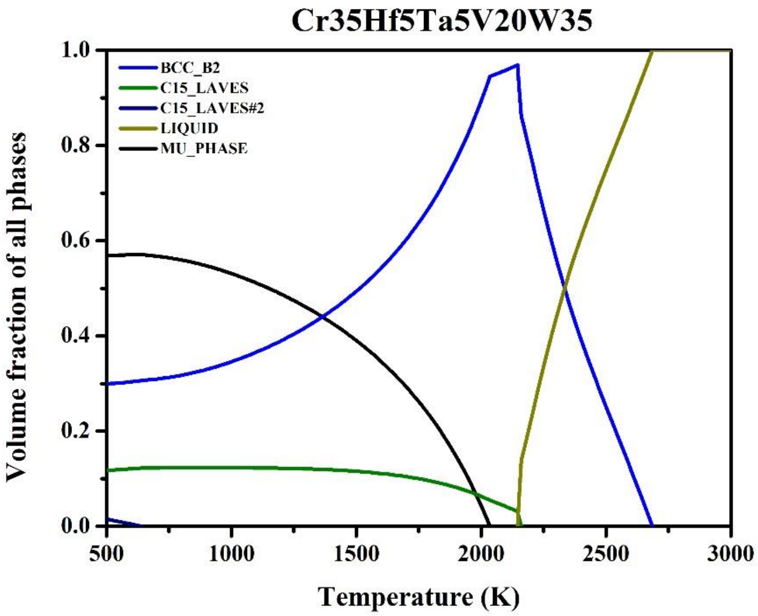

Supplementary Figure 8: Calphad phase stability diagram for Cr35Hf5Ta5V20W35

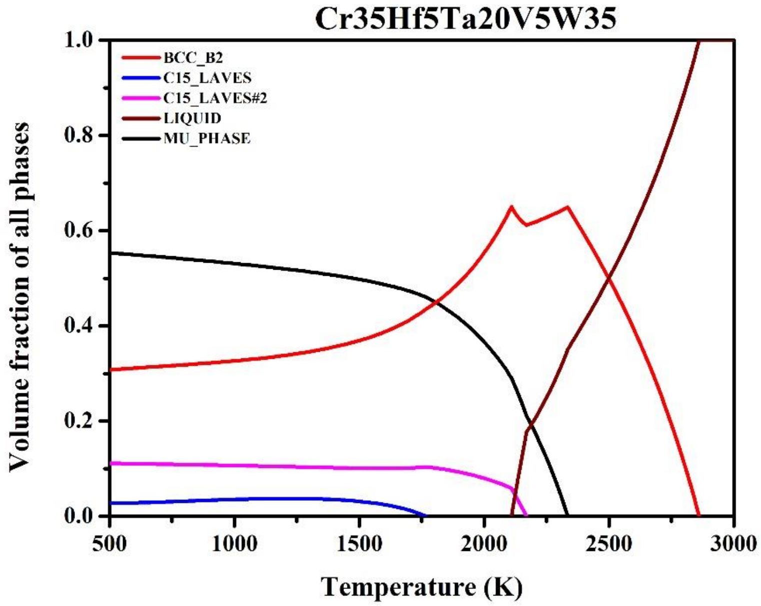

Supplementary Figure 9: Calphad phase stability diagram for Cr35Hf5Ta20V5W35

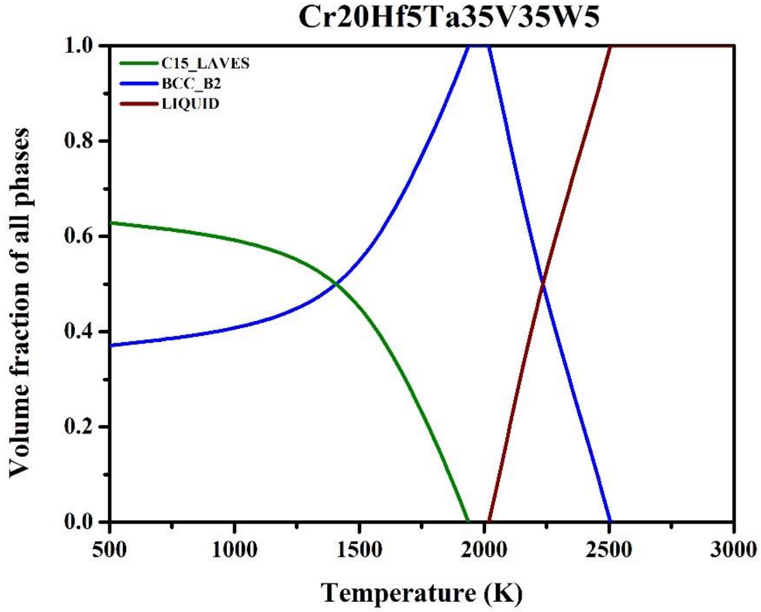

Supplementary Figure 10: Calphad phase stability diagram for Cr20Hf5Ta35V35W5

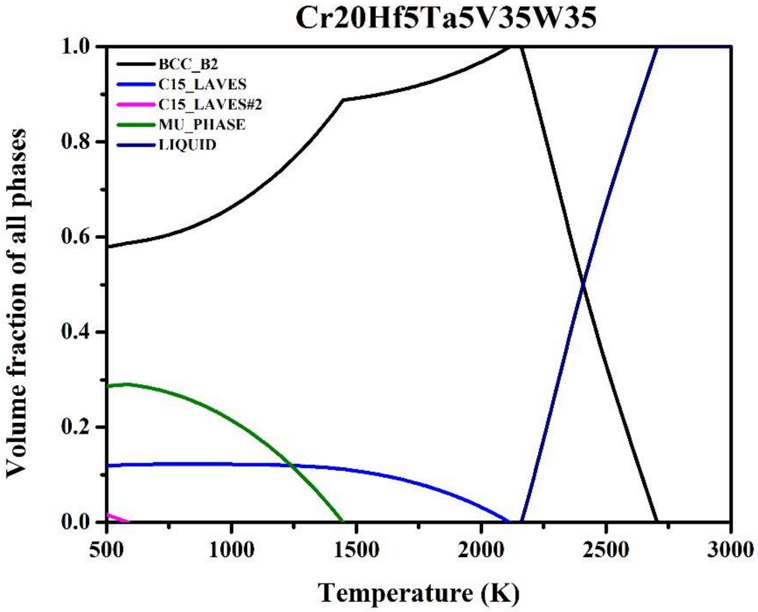

Supplementary Figure 11: Calphad phase stability diagram for Cr20Hf5Ta5V35W35

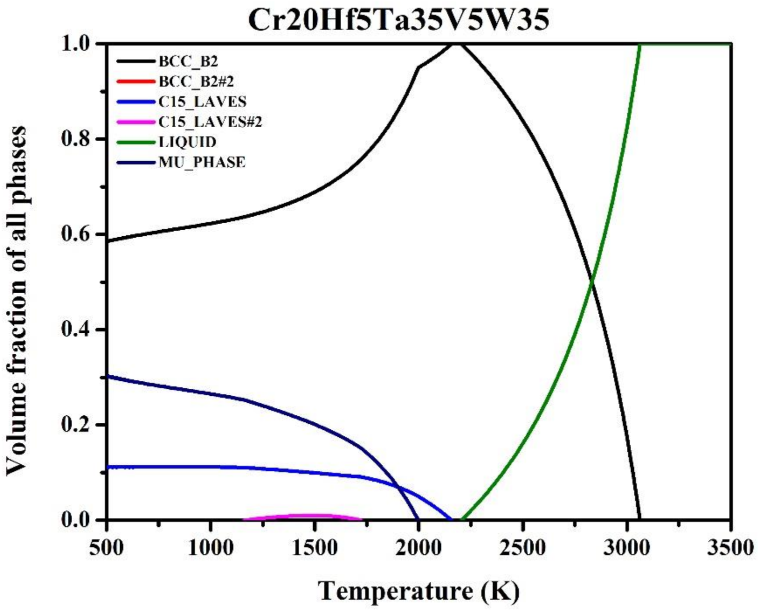

Supplementary Figure 12: Calphad phase stability diagram for Cr20Hf5Ta35V5W35

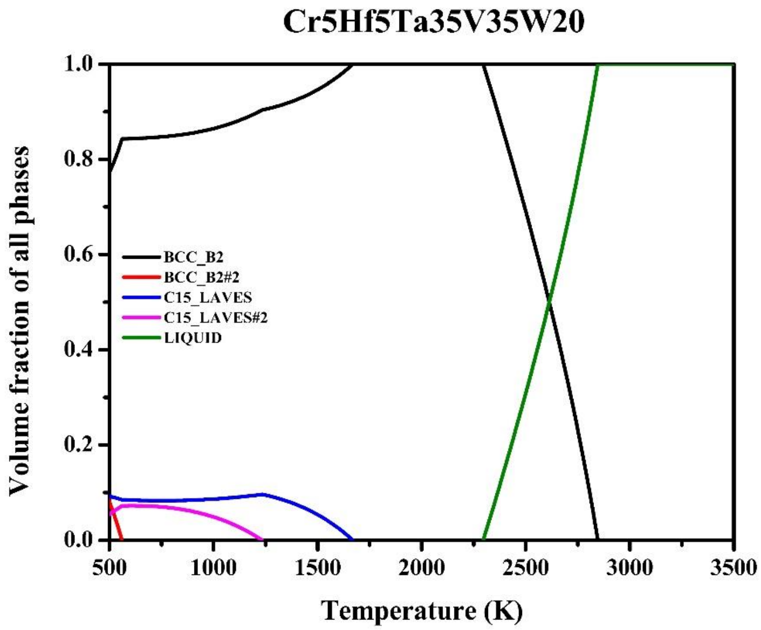

Supplementary Figure 13: Calphad phase stability diagram for Cr5Hf5Ta35V35W20

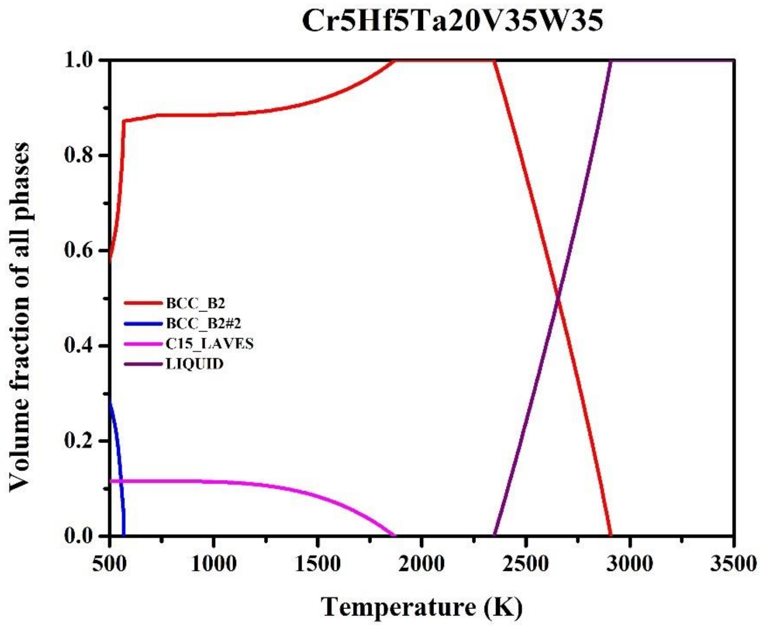

Supplementary Figure 14: Calphad phase stability diagram for Cr5Hf5Ta20V35W35

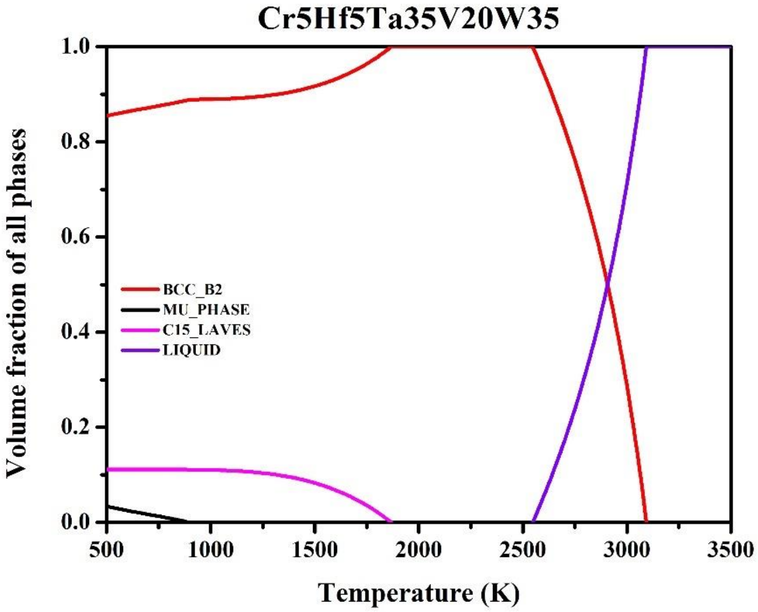

Supplementary Figure 15: Calphad phase stability diagram for Cr5Hf5Ta35V20W35

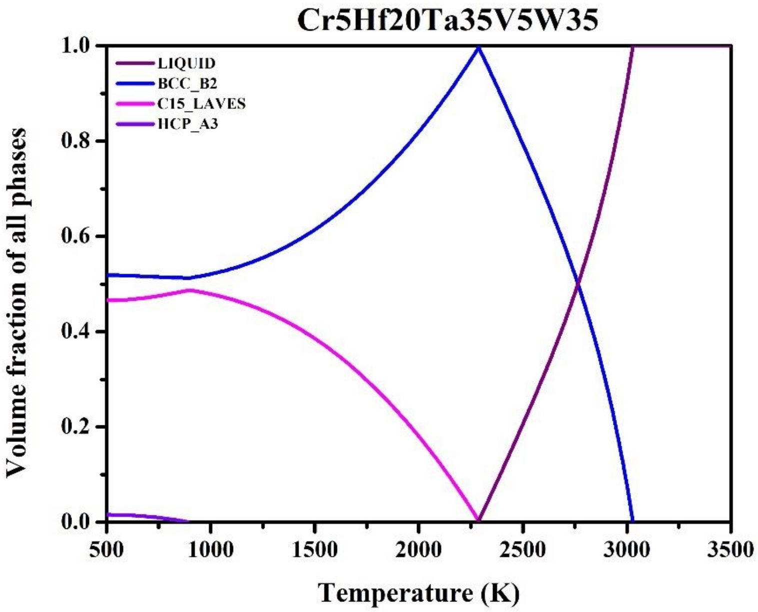

Supplementary Figure 16: Calphad phase stability diagram for Cr5H20Ta35V5W35

We also tried to maximize Hf to 10%. For 10% Hf, few compositions (with minimizing Cr compositions) satisfied the single phase criteria as shown in Supplementary Table 4. However, one composition demonstrated large single phase region in CALPHAD.

Supplementary Table 4: Thermophysical parameter calculations for the W-Ta-Cr-V-Hf system for 10% Hf.

Alloy formula	Density	VEC	ΔS mix	Tm (K)	δ[1]	ΔH mix[2]	Ω	solid solution
**Cr5Hf10Ta35V15W35**	**15.17**	**5.3**	**-11.64**	**3132**	**5.57**	**-5.77**	**6.32**	**yes**
**Cr5Hf10Ta15V35W35**	**13.06**	**5.3**	**-11.64**	**2910**	**5.92**	**-3.91**	**8.67**	**yes**
**Cr5Hf10Ta35V35W15**	**12.54**	**5.1**	**-11.64**	**2829**	**6.07**	**-5.30**	**6.21**	**yes**

The phase stability diagram of the three compositions in table 3 are shown below:

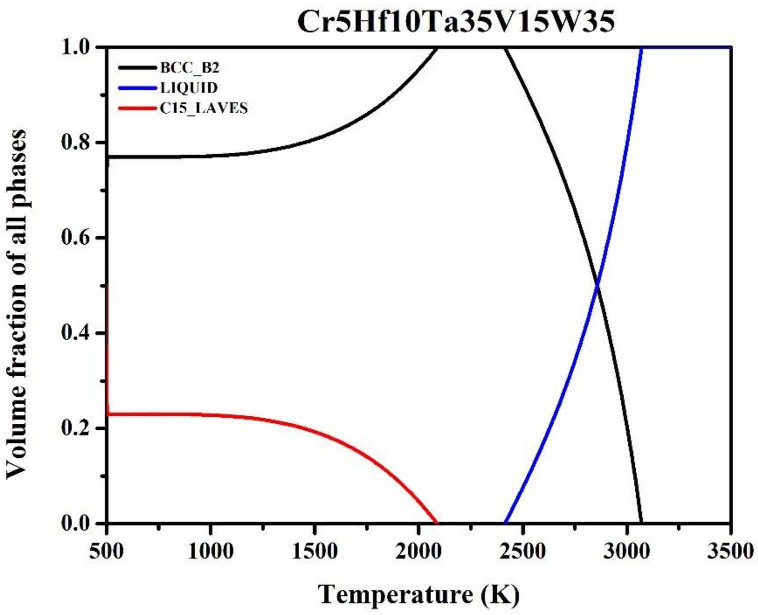

Supplementary Figure 17: Calphad phase stability diagram for Cr5Hf10Ta35V15W35

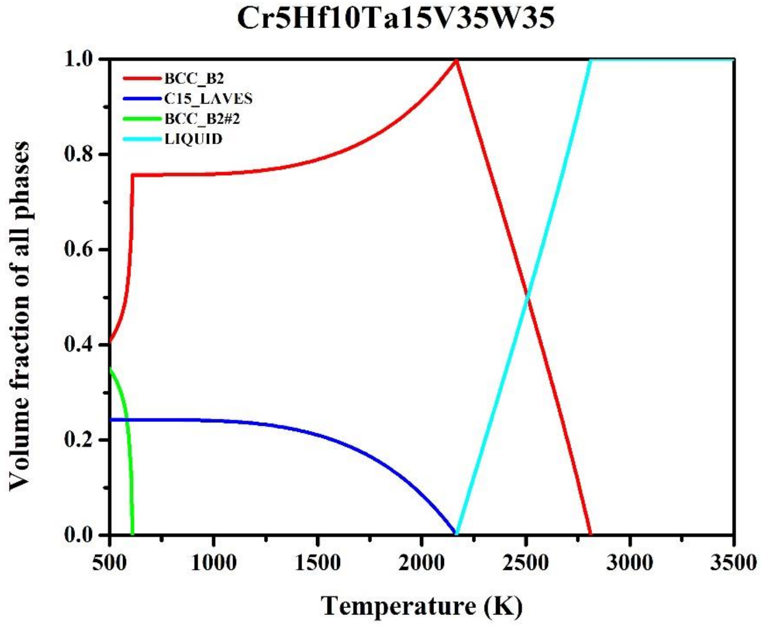

Supplementary Figure 18: Calphad phase stability diagram for Cr5Hf10Ta15V35W35

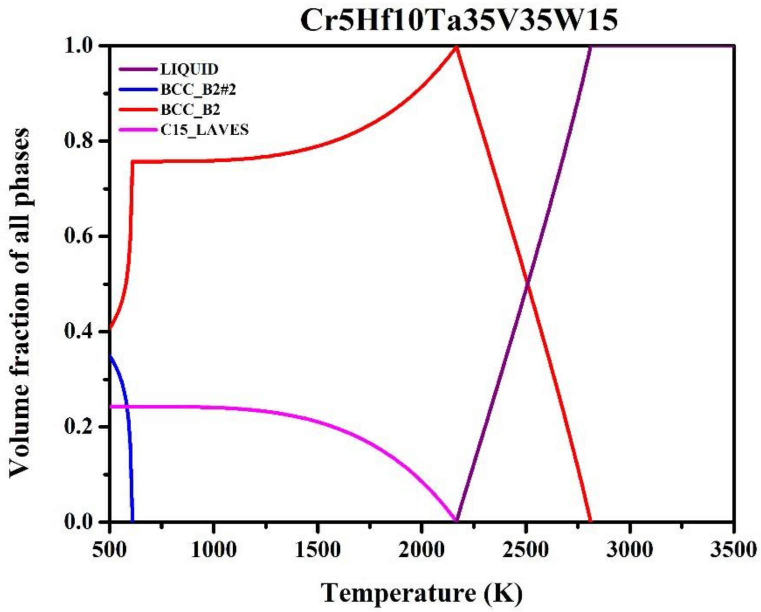

Supplementary Figure 19: Calphad phase stability diagram for Cr5Hf10Ta35V35W15

Therefore, only 4 compositions are predicted (from the thermophysical parameter calculations and CALPHAD) to have single BCC phase which are:

**Cr5Hf5Ta35V35W20**
**Cr5Hf5Ta20V35W35**
**Cr5Hf5Ta35V20W35**
**Cr5Hf10Ta35V15W35**

We then synthesize two films (using magnetron deposition) which are the ones studied and illustrated in the paper. The phase stability diagrams of these compositions (which satisfy the thermophysical parameter criteria and CAPLHAD) are shown below:

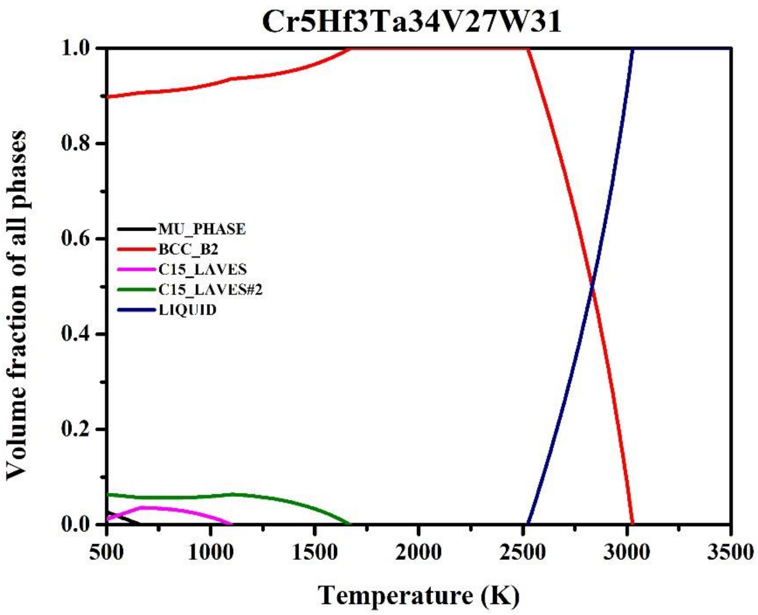

Supplementary Figure 20: Calphad phase stability diagram for Cr5Hf3Ta34V27W31

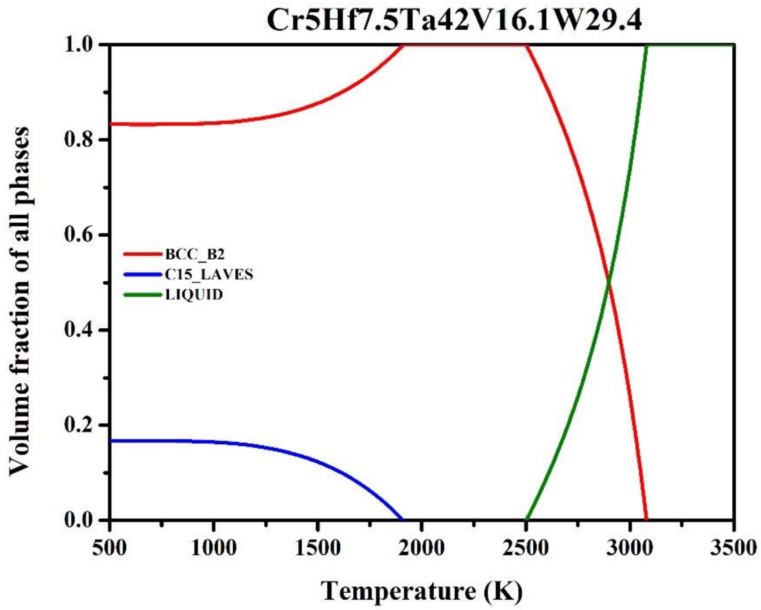

Supplementary Figure 21: Calphad phase stability diagram for Cr5Hf7.5Ta42V16.1W29.4

Supplementary References

1. Ziegler JF, Ziegler MD, Biersack JP. SRIM–The stopping and range of ions in matter (2010). Nuclear Instruments and Methods in Physics Research Section B: Beam Interactions with Materials and Atoms. 2010;268(11):1818-1823.
2. Jung P. 1.7 Production of atomic defects in metals by irradiation. Atomic Defects in Metals: Springer. p. 6-7.
3. Senkov ON, Miracle DB. Effect of the atomic size distribution on glass forming ability of amorphous metallic alloys. Materials Research Bulletin. 2001;36(12):2183-2198.
4. De¸bski A, De¸bski R, Gasior W. New features of Entall database: Comparison of experimental and model formation enthalpies. Archives of Metallurgy and Materials. 2014;59(4):1337-1343.
