# Supplementary material for: A quinary WTaCrVHf nanocrystalline refractory high-entropy alloy withholding extreme irradiation environments
Source: Nat Commun. 2023 May 2;14:2516. doi: 10.1038/s41467-023-38000-y (PMC10154406; doi:10.1038/s41467-023-38000-y)
Supplement: Supplementary file 3 — Description of Additional Supplementary Files [file 41467_2023_38000_MOESM3_ESM.pdf]

Description of Additional Supplementary Files

File Name: Supplementary Movie 1
Description: Dual beam irradiation of $W_{29.4}Ta_{42}Cr_{5.0}V_{16.1}Hf_{7.5}$ at 1173 K
